# Effect-Based Tools for Monitoring and Predicting the Ecotoxicological Effects of Chemicals in the Aquatic Environment

**DOI:** 10.3390/s120912741

**Published:** 2012-09-18

**Authors:** Richard E. Connon, Juergen Geist, Inge Werner

**Affiliations:** 1 School of Veterinary Medicine, Department of Anatomy, Physiology and Cell Biology, University of California, Davis, CA 95616, USA; 2 Aquatic Systems Biology Unit, Department of Ecology and Ecosystem Management, Technische Universität München, Mühlenweg 22, D-85354 Freising, Germany; 3 Swiss Centre for Applied Ecotoxicology Eawag-EPFL, Überlandstrasse 133, CH-8600 Dübendorf, Switzerland

**Keywords:** bioassays, biomarkers, toxicity identification, ecosystem effects, ecotoxicogenomics, ecotoxicology, aquatic toxicology, ecological monitoring, adverse outcome pathways, systems biology

## Abstract

Ecotoxicology faces the challenge of assessing and predicting the effects of an increasing number of chemical stressors on aquatic species and ecosystems. Herein we review currently applied tools in ecological risk assessment, combining information on exposure with expected biological effects or environmental water quality standards; currently applied effect-based tools are presented based on whether exposure occurs in a controlled laboratory environment or in the field. With increasing ecological relevance the reproducibility, specificity and thus suitability for standardisation of methods tends to diminish. We discuss the use of biomarkers in ecotoxicology including ecotoxicogenomics-based endpoints, which are becoming increasingly important for the detection of sublethal effects. Carefully selected sets of biomarkers allow an assessment of exposure to and effects of toxic chemicals, as well as the health status of organisms and, when combined with chemical analysis, identification of toxicant(s). The promising concept of “adverse outcome pathways (AOP)” links mechanistic responses on the cellular level with whole organism, population, community and potentially ecosystem effects and services. For most toxic mechanisms, however, practical application of AOPs will require more information and the identification of key links between responses, as well as key indicators, at different levels of biological organization, ecosystem functioning and ecosystem services.

## 1. Introduction

“*People often ask, ‘What is the single most important environmental problem facing the world today?’ The single most important problem is our misguided focus on identifying the single most important problem! … because any of the dozen problems, if unsolved, would do us great harm and because they all interact with each other.*” Jared Diamond [1].

The field of ecotoxicology studies the effects of anthropogenic chemicals on ecosystems at different levels of biological organization, from the molecular and cellular level to entire ecosystems. Aquatic ecosystems are often impacted by chemical pollution, originating from municipal and industrial wastewater effluents (point sources), airborne deposition as well as runoff from urban and agricultural areas (diffuse sources). Ecotoxicology is closely related to, but sometimes distinguished from stress ecology, which considers a broader range of natural stressors such as the effects of temperature or oxygen depletion on individuals, populations and communities, however these parameters directly impact on toxicity. There is further separation between ecotoxicology and environmental toxicology, which is often rather artificial, with a tendency of environmental toxicology being more focused on the level of individual organisms, including humans, or cells, and ecotoxicology being more focused on the level of communities and ecosystems.

One of the core missions of ecotoxicology is to understand the mechanisms by which contaminants perturb normal biological performance (their mode of action), in order to develop appropriate measures to prevent adverse outcomes resulting from environmental contaminants. There is a wide range of possible contaminant effects that can compromise the ecological fitness of individual organisms or populations. Ultimately, the impact of a toxic contaminant or contaminant mixture depends on the relative sensitivity of a species, community or ecosystem, and the intensity and timing of exposure. Acutely toxic events—most notably fish kills—which were relatively common a few decades ago, are now rarely observed in most industrialised countries, however, even sublethal toxicity can lead to severe impacts on entire populations.

Of the vast number of substances that have been introduced to aquatic systems around the world, a number posing serious environmental threats have primarily been identified either through single-species toxicity testing in the laboratory, or they have been brought to light as a result of observing biological effects *in situ*. Examples include dichlorodiphenyl-dichloroethylene (DDE) and eggshell thinning in birds [2], sex changes in freshwater fish associated with endocrine disrupting chemicals, EDCs [3], and tumors in marine fish associated with polycyclic aromatic hydrocarbon (PAH) accumulation [4]. These examples highlight the need for techniques that not only detect overt damage to organisms exposed to pollutants, but also the less obvious biochemical and physiological impairment that might ultimately result in ecological damage [5].

With more than 67 million organic and inorganic substances known to date [6], monitoring and assessing effects of chemical pollution necessarily faces great challenges. In light of recent legislation by the European Union, such as Registration, Evaluation, Authorisation and Restriction of Chemical substances (REACH) and the Water Framework Directive (WFD), ecotoxicology needs to become more proactive. Consequently, the development of reliable predictive tools to assess the impacts of chemicals as well as robust and sensitive retrospective tools to monitor water quality will be important for advancing this field.

## 2. Current Approaches

Toxic substances can cause effects at different levels of biological organisation, from molecular to ecosystem levels. Of primary concern is the protection of aquatic organisms at the population or ecosystem level; it is therefore important to bridge the gap between the relatively short-term (acute) effects that can realistically be quantified in laboratory or field experiments, and longer-term (chronic) ecological effects.

### 2.1. Risk Assessment

**Figure 1.**
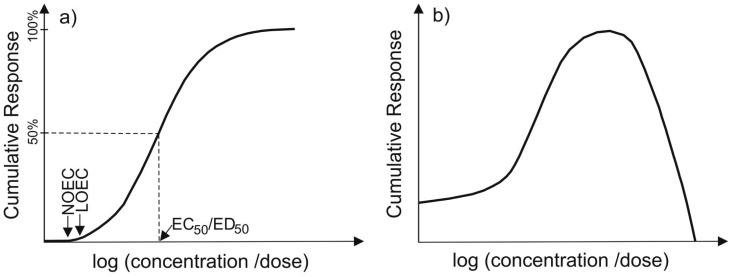
Different types of response to toxicant exposure: (**a**) classical dose-response (greater effect with increasing dose) and (**b**) example of a nonmonotonic response (greater effect with decreasing dose after peak, referring to sublethal effects).

Effects of chemicals on aquatic biota depend on the concentration, toxicity, solubility, bioavailability, and duration of exposure, as well as the sensitivity of the exposed organisms. Consequently, acute (typically <7 days) and chronic (typically >7 days), lethal and sublethal effects are distinguished. Depending largely on water solubility, the pathways of contaminant uptake (e.g., via gill tissue or via food uptake) may greatly alter their ecotoxicological effects (e.g., [7]). Indirect effects such as depletion of food organisms can significantly impact species at higher trophic levels [8,9]. Such responses do not always follow classical dose-response curves [10], therefore nonmonotonic dose response patterns (*i.e.*, effects occurring only at low doses; Figure 1) must be considered and integrated into the derivation of environmental quality standards (EQS) or criteria, and risk assessment.

For individual chemicals, environmental risk assessment is typically achieved by combining hazard and exposure assessments (e.g., [11]). Hazard assessment refers to the determination of the toxicological effects of a substance on biota, including the analysis of adverse effects at different exposure concentrations. Of critical importance is the level or concentration at which no effects are observed (no observed effect level, NOEL, or, no observed effect concentration, NOEC), as well as the concentration or dose at which 50% of an exposed population are affected (effect concentration/dose EC_50_ or ED_50_, for mortality as endpoint: lethal concentration or dose, LC_50_ or LD_50_, Figure 1(a)).

Effect (or hazard)-based EQS or criteria can be derived from such effect data, which typically includes a safety factor based on the availability of toxicity data, *i.e.*, if data is available for three or more taxa (Table 1, [12]). Where sufficient effect data are available, species sensitivity distribution (SSD) curves are used to derive the exposure concentration of a given chemical that is protective of 95% of species. Environmental quality standards have been used since the 1970s, after the US Water Quality Act of 1965 required the development of numeric criteria for the protection of human health. With the enactment of the US Clean Water Act in 1977, EQS became valuable tools to assess the risk of individual chemicals in the aquatic environment. In the EU the derivation of EQS is driven by the WFD [13,14] which establishes a legal framework to protect and restore clean water across Europe and ensure its long-term, sustainable use.

**Figure 2.**
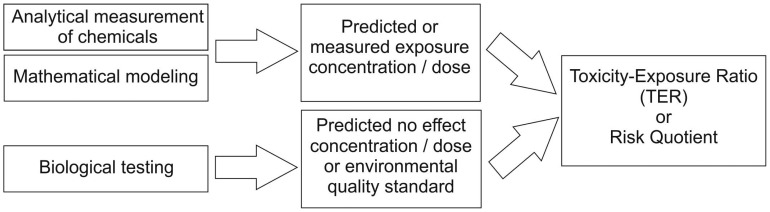
Determination of toxicity-exposure ratios (TER) or risk quotients by combining data on exposure scenarios with expected biological effects and/or environmental standards.

Exposure assessment refers to the determination or prediction of the extent, frequency and duration at which biota are being exposed to a chemical in nature. Mathematical exposure models take into consideration the environmental fate, transport, and bioavailability of the compound; where analytical data exist, the median concentration or the 90th percentile is used. Typically, persistent substances with high bioavailability increase the risk of exposure, however, when less persistent substances are continuously introduced into an ecosystem (e.g., via point sources), exposure scenarios resemble those of persistent chemicals (e.g., contaminants within wastewater treatment effluents). Such continuously introduced substances are sometimes referred to as “pseudo-persistent” contaminants. The ratio of hazard to exposure values, so-called toxicity/exposure ratios (TER) or risk quotients, are determined for both short- and long-term exposures; they should not exceed certain trigger values (Figure 2).

Risk assessment is an important tool for achieving the protection goals of current regulations, however, the ability to perform risk assessments for all chemicals of interest is—apart from being time consuming and costly—often hampered by the lack of effect data. In addition, their usefulness in assessing the combined effects of chemicals and other stressors (physical, biological) and of chemical mixtures is limited. In natural settings, the exposure of organisms and communities to mixtures of chemical compounds must be considered to be the most common exposure scenario. For example, a US-wide survey in the 1990s showed that over 50% of more than 4,000 stream water samples contained six or more pesticides [15]. Treated municipal wastewater contains a multitude of organic chemicals, including pharmaceuticals, hormones, and pesticides [16,17], which are continuously introduced into aquatic ecosystems. Simultaneous exposures to multiple chemicals can exert additive, synergistic, or antagonistic effects; equal to, greater than, or less than the sum of the independent effects of each contaminant, respectively.

**Figure 3.**
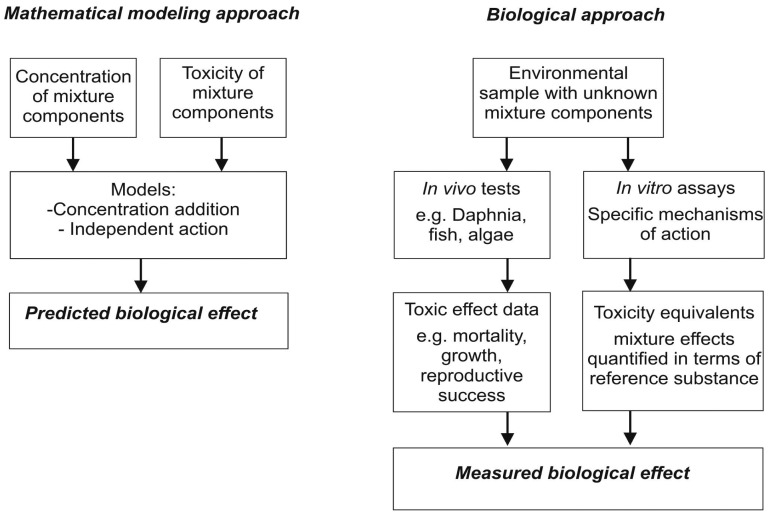
Two approaches to mixture toxicity assessment: effect prediction using mathematical modeling and single–substance toxicity data, and effect measurement using effect-based biological tests.

To date, however, regulatory requirements on risk assessment of chemicals are largely based on individual substances. It was only within the last decade that broad interest in the need for assessing the risks of combined exposure to multiple substances started to mount [18]. Currently available approaches for assessing the toxicity of chemical mixtures include theoretical models, each with different levels of data requirements [19] and biological tools [20] (Figure 3). Considering that it is not possible to analyze, detect and quantify all substances that are present in water bodies, including emerging pollutants and transformation products, the importance of integrating biological tools into monitoring efforts becomes apparent. Effect-based tools can be useful in the development of rational and cost-effective monitoring programs, to improve the environmental relevance of the assessment, and to link ecological and chemical information.

### 2.2. Biotests

Long before the onset of environmental regulation, biological tools based on indicator species were used to detect environmental hazards, such as the “canary in the coal mine” used to warn miners of dangerous levels of carbon monoxide and methane. Standardized biological test methods to measure water quality developed quickly after the US Environmental Protection Agency (US EPA) initiated a national policy in 1984 to control toxic substances based on a water quality approach aimed at the protection of environmental health using biotests. In the US (and later elsewhere), the issuance of permits for effluent discharges into surface waters was subsequently tied to whole effluent testing using standardized toxicity tests (http://water.epa.gov/scitech/methods/cwa/wet/index.cfm). Such tests enable the direct measurement of toxicity independent of the number of causative chemicals or mixture effects. Biotests can measure integrative toxic effects and provide the data needed for the derivation of EQS and risk assessment. To date, however, most environmental monitoring programmes rely primarily on the analysis of chemical substances in water column, sediment and biota. The resulting assessment is then performed on a single-substance basis.

**Figure 4.**
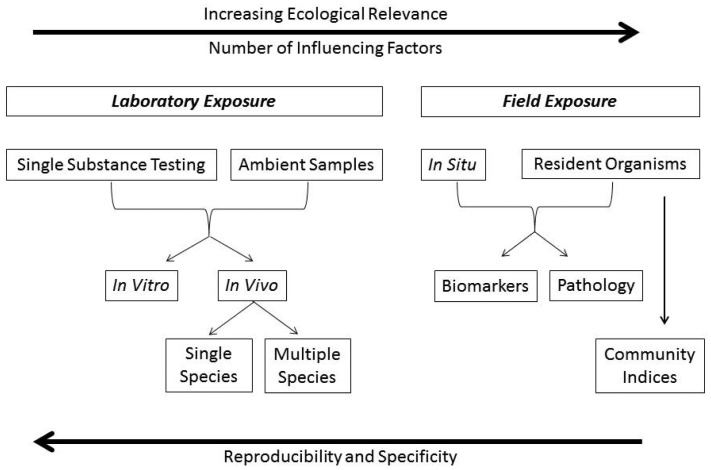
Different biological approaches exist for measuring toxicity of chemicals and their effects in the aquatic environment. With increasing ecological relevance the reproducibility, specificity and thus suitability for standardization of methods diminishes. Biomarkers can help bridge this gap as they can be effect- and/or chemical specific.

Biotests can target different levels of biological organization, from the level of molecules and cells, to tissues and organs, individuals, populations and communities. Depending on the objectives of an investigation, biotests can be performed under standardized conditions in laboratories using single cell systems (*in vitro*), organisms (*in vivo*) or simple communities (micro-, mesocosms), or—where the focus is on ecological relevance—in the field, by means of *in situ* exposures, health assessment of resident organisms using biomarkers (molecular, biochemical, cellular and/or physiological alterations) and (histo-) pathological evaluation, and/or community indices (Figure 4).

#### 2.2.1. *In Vitro* Tests

There is a wide range of possible contaminant effects that are not lethal, but can compromise the ecological fitness of individual organisms or a population. They can severely reduce ecological fitness and ultimately survival, since the individual must be able to successfully compete with others for food, avoid predation, reproduce, and cope with pathogens and other environmental stressors. Such effects are not easily detected and can act for long periods of time before being recognized.

Effect (mode-of-action)-specific *in vitro* tests (Table 2) allow the rapid and sensitive detection of chemical activity in biological systems. They generally measure effects at the cellular level and are designed for high throughput applications in the laboratory, for example the screening of environmental samples for the identification of pollution “hot spots”. While they have the great advantage of measuring the combined effects of chemical mixtures with the same mode-of-action, the predictive power of most *in vitro* assays for deleterious effects at higher levels of organization is still limited. In addition to environmental samples (e.g., soil, water, sediments, tissue extracts), some of the currently available *in vitro* systems can also be applied as biomarker-assays on enzymes, cells and tissues sampled from living organisms

Where scientific knowledge is abundant, and linkages between effects at the cellular level and population responses have been established, *in vitro* tests can be effectively used for monitoring of water quality. Prominent examples are test systems for estrogenic activity. Recombinant cell culture or yeast based systems such as Chemical Activated Luciferase Gene Expression (CALUX, [21,22]) and Yeast Estrogen Screen (YES) are based on a reporter gene approach and respond to substances that bind to the human estrogen receptor [23]. It has been shown, that estrogens and other hormonally active chemicals (or EDCs) interfere with the normal functioning of the endocrine system. Exposure to extremely low concentrations can impede gonadal function, reduce fertilization success, decrease fecundity and alter mating behavior [24,25] in aquatic species. One of the more potent estrogens found in surface waters is 17α-ethinylestradiol (the synthetic estrogen used in birth-control pills). This compound, like natural estrogens, is not completely removed by sewage treatment plants [26], and can cause the collapse of fish populations at trace concentrations of <6 ng/L [27]. Other known EDCs include industrial chemicals and waste products such as polychlorinated dibenzodioxins, furans and PCBs, organochlorine and pyrethroid pesticides (and their metabolites), surfactants such as nonylphenol polyethoxylates used in pesticide formulations, and phthalates used in plastic products. Despite decades of research on this topic, however, no water or sediment quality criteria currently exist for protecting human and aquatic life against endocrine system disruption and its related effects, and existing *in vitro* biotests have not yet been standardized.

#### 2.2.2. *In Vivo* Tests

##### Laboratory Tests with Single Species

*In vivo* bioassays are tests in which whole living organisms are exposed to spiked or ambient samples (water, sediment) or extracts from these samples. Standardized bioassays rely on measuring responses in readily available model species which may not be representative of other more vulnerable species, however, they allow for the quantification of chemical-caused toxic effects that are separated from other environmental stressors (*i.e.*, under conditions that are otherwise ideal for the test organisms). There are two distinct areas where such bioassays are used routinely due to legal requirements: (1) single substance testing for the purpose of product registration, and (2) the testing of environmental (effluent, dredged sediments) samples. In some countries they are also used for monitoring water quality (e.g., US, Czech Republic, France). The two applications require standardized testing procedures so that data produced are comparable, and meet strict quality standards. Internationally, the Organization for Economic Co-operation and Development (OECD) and International Organization for Standardization (ISO) are the most important bodies for development, validation and standardization of analytical as well as effect-based test methods. Whereas OECD is focused on test methods for single substance testing (“toxicity tests”), ISO is dedicated to the environmental aspects of water quality control. Other important bodies for validation and standardization of bioassays/toxicity tests are the American Society for Testing and Materials (ASTM) and the US EPA.

Bioassay batteries used in environmental monitoring are often based on the concept of a simple food chain using at least three species from different trophic levels: typically a primary producer (e.g., green algae), a detritivore or filter feeder (e.g., waterflea), and a consumer (e.g., larval fish). Table 3 summarizes species, exposure periods, test types and endpoints used for standardized *in vivo* bioassays in freshwater. The need for a reduction in the numbers of vertebrate animals used in testing for ethical reasons has prompted the switch to a 48-h fish egg test (*Danio rerio*) in the EU (ISO 15088, 2007).

The definitions for acute and chronic tests vary between different organizations. In their guidelines for the evaluation of acute toxicity, the United Nations [42] define short term or acute tests (hours to a few days) as those which determine an LC_50_ (concentration causing mortality in 50% of exposed organisms) or EC_50_ (concentration causing an adverse effect in 50% of exposed organisms).

Chronic toxicity is determined within a test period related to the life cycle of the organism (generally days, weeks or months). In this case, sublethal effects are preferred to mortality as endpoint, and the no observed effect concentration (NOEC) is determined rather than an LC/LD_50_ (lethal concentration/lethal dose at which 50% mortality is observed). The use of NOEC in risk assessment has lately received considerable criticism (e.g., [43]).

Where toxicity identification evaluation (TIE) protocols exist (e.g., tests with *Ceriodaphnia dubia*, [44,45]), the identification of the dominant toxicant(s) or toxicant group(s) is possible. The TIE approach has its origin in whole effluent testing, which focuses on the question, whether an effluent will cause adverse effects on aquatic organisms when emitted to the environment. In the case that effects are detected in whole organisms under realistic exposure conditions, TIE should help to characterize and identify the cause of the measured effect. Thus, TIE applies *in vivo* bioassays and avoids extraction and pre-concentration steps as far as possible; it has been shown to be a powerful approach to characterize highly contaminated sites with acute toxicity caused by compounds that are well characterized (e.g., [46]). Where toxicity is below the acute level, however, currently available TIE procedures are unable to yield conclusive information. Effect-directed analysis (EDA) combines bioassays, physico-chemical fractionation procedures and chemical analysis in a sequential procedure to identify unknown toxicants in complex environmental samples (e.g., [47,48]). EDA can be based on any toxicological, ecotoxicological or biological endpoint that can be detected and quantified with sufficient throughput, generally *in vitro* assays where specific effects can be quantified (Table 2). Emerging tools such as ecotoxicogenomics techniques (see Section 3) may be well-suited in this context to identify important pathways of toxicity, complement existing bioassays and/or serve as diagnostic tools.

##### Laboratory Tests with Multiple Species

Environmental and ecological relevance of ecotoxicological testing can be increased if effects on multiple species, mimicking natural communities or simple food webs, are considered. Such an approach can consequently be useful for reducing assessment and risk factors (Table 1). In contrast to combining separate single-species test data for several species, simultaneous exposure of multiple species also integrates interaction among species such as competition and predation. Laboratory tests which simultaneously use multiple species try to find a balance between standardization and natural variation of abiotic conditions, e.g., temperature, light and pH. Theoretically, test species should be selected according to their abundance or importance (keystone species) in the type of habitat for which ecotoxicological effects are being assessed. For practical reasons, however, most studies rely on simplified food chains, including primary producers (different algae or macrophytes), consumers (e.g., daphnids, snails, grazing insect larvae) and predators (e.g., beetles, fishes). Due to size constraints, fish are only used in few test systems despite the fact that they can play important roles in top-down control. Guidance documents (Guidance Document on simulated freshwater lentic field tests; outdoor microcosms and mesocosms) and standard protocols (e.g., OECD Series on Testing and Assessment Number 53 [49]) for tests with multiple species have been proposed. In line with field methods, laboratory tests with multiple species rely on a retrospective analysis of effects, which is often limited by large variability and relatively slow community responses at low exposure concentrations or doses.

##### Field Methods

Toxicity endpoints that can be measured in laboratory tests ignore the additional physical and biological stressors organisms encounter in their natural environment. Likewise, the continuous exposure to ambient water samples or a constant concentration of single chemicals often does not reflect intermittent or pulse exposure scenarios common in natural settings. Measurement of exposure or effect indicators in field-collected or *in situ*-exposed organisms, or biomarkers, are therefore far more ecologically meaningful than laboratory tests, where studies are focused on monitoring the health of wild populations.

###### *In situ* Tests

*In situ* tests represent an intermediate step between the controlled conditions of laboratory assays with model species and the environmental realism of field monitoring [14]. They integrate the combined effects of complex environmental conditions and potentially variable exposure to toxic chemicals, thus facilitating the assessment of toxicity in the field. For example, toxicity measured *in situ* can be much higher than predicted from analytical measurements alone due to unknown (and therefore un-quantified) contaminants and stressors that might be acting simultaneously [50]. However, site-specific physical factors may influence results [51]; thus *in situ* studies require careful planning and can require significant resources. In addition, *in situ* systems are prone to extreme events and other disturbances, including floods and vandalism. It is therefore crucial to clearly define research questions and hypotheses before conducting *in situ* studies.

Methods for *in situ* studies are diverse and generally not standardized. They include caged studies [51], bypass (flow-through) systems [52], and transplanted organism studies [53], usually conducted with fish and/or macroinvertebrates. A Pellston Workshop on this topic (*in situ* measures of ecological effects) was conducted by the Society of Environmental Toxicology and Chemistry in 2004 [54]. Test organisms may be site-relevant model species or key resident species [14,55]. When designing *in situ* studies the following considerations are of primary importance: (1) test organisms must not be stressed by their enclosures; (2) chemical exposure should be characterized to identify the causes of toxic effects; and (3) habitat conditions of reference/control sites must be comparable to experimental sites. To interpret results obtained from *in situ* studies in an ecological context, they should be made part of a weight-of-evidence approach [56], and linked to protection goals [55].

###### Biomarkers: Monitoring Organism Exposure and Health

Biomarkers are important tools to measure the sublethal effects of chemicals in wild organisms. Biomarkers are analyzed directly in cells and tissues of exposed organisms, and are traditionally defined as molecular, biochemical, cellular and physiological alterations caused by external stressors [57]. Biomarkers of exposure indicate exposure reflective of the internal concentration of chemical(s) or metabolite(s), and can be applied as screening tools for specific chemical groups, for example the proteins metallothionein or CYP1A as indicators of elevated concentrations of bioavailable heavy metals or PAHs/PCBs, respectively [58,59]. Biomarkers of effect indicate deleterious effect or functional changes at the organismal level. These can provide early warning signals of declining organism health or environmental disturbance. Among them is vitellogenin, a precursor of egg yolk protein, whose linkage to endocrine disruption and reproductive effects is well established and standardized procedures are under development (Table 4). Its production is enhanced in response to chemicals with a specific (estrogenic) mode of action and has been linked to effects at higher levels of biological organization [27]. Histopathological examination, especially of fish, has long been used for the assessment of organism health, and has been shown to be a reliable tool for evaluating the effects of environmental contamination at the tissue and organ level [60].

For environmental managers it is of great importance to discover effects related to chemical substances before significant effects at the population level can occur; however, very few biomarkers are currently understood well enough to provide conclusive evidence of environmental stressor impacts on their own [61], especially because response curves are often nonmonotonic, and physical environmental stressors can influence the response [62]. For identifying pollution “hot spots” or monitoring organism health, it is therefore important to employ batteries of sensitive biomarkers covering a broad range of effects in a weight of evidence approach [62,63]. This is why ecotoxicogenomic approaches may soon become the biomarker tools of choice, as a broad spectrum of response parameters can be quantified in a single sample, and methods are easily transferrable to different species (see Section 3). Already the measurement of some well-known biomarkers such as CYP1a, vitellogenin and aromatase is conducted at the mRNA rather than the protein level in many investigations (Table 4).

###### Community Indices

Organism communities are at a level of biological organization that represents ecosystem integrity since they result from the combined effects of physical, chemical and biological stressors acting in a system. Community responses can be described in terms of changes in structure (number, abundance and diversity of species), and function or tolerance to pollutants over time. Respective indices are based on the concept that sensitive components of the exposed community will be replaced by more tolerant species during exposure. While community indices have high ecological relevance, the ability to identify the underlying causes/stressors of negative effects is limited, as communities may respond similarly to different pressures, such as organic enrichment, eutrophication, toxic chemicals or habitat degradation.

Among aquatic community indices, one of the most widely applied is the index of biological integrity (IBI; [79,80]). IBIs, which are region-specific, have been developed for fish, algae, macroinvertebrates, pupal exuvia (shed skins of chironomidae), vascular plants, and combinations of these. In Europe, the saprobic index for benthic stream macroinvertebrate communities [81] has been widely used for the assessment of organic water pollution. A relatively recent development of the community approach, is the SPEcies At Risk (SPEAR) index [82]. It characterizes stream macroinvertebrate communities according to their sensitivity to insecticides. The SPEAR bioindicator system has been shown to be highly sensitive, relatively independent of confounding factors [82,83] and applicable across different biogeographical regions [84].

Most aquatic plant groups, such as macrophytes, filamentous algae, mosses, periphyton and phytoplankton, as well as bacteria have been used as indicators of water pollution and eutrophication (e.g., [85,86]). The pollution induced community tolerance (PICT; [87,88]) approach directly quantifies the level of adaptation in natural periphyton, phytoplankton, bacteria or nematode communities to toxic chemicals. Tolerance development can be, for example, measured as a shift in the effect concentration (e.g., EC_50_) of a specific chemical, which is determined in a short-term toxicity test.

## 3. Promising New Approaches and Tools

Forty four years after the word “ecotoxicology” was coined by René Truhaut in 1969 [89], technological and computational advances have enabled a number of assessment methodologies to arise, as well as new technology to continually evolve; e.g., gene expression assessments using differential display reverse transcription PCR (dd-rtPCR) to whole transcriptome assessments using microarrays and more recently direct sequencing (RNASeq), or single protein analysis using western blots, to high-throughput proteomics. The rapid development of new technologies such as transcriptomics, proteomics and metabolomics are revolutionizing ecotoxicology [90], however, with this development, the volume of generated data has also increased exponentially, often beyond the capacity of what can realistically and coherently be analyzed. These “new” technologies have had a significant impact on medical research and are gradually being incorporated into ecotoxicological studies. This is a challenging task since medical research primarily focuses on responses at the level of the individual and one species, whereas ecological research attempts to utilize these systems to encompass populations, species communities and ecosystems. Nevertheless, a number of successful ecological case studies have demonstrated the usefulness of novel technologies, in complement with traditional approaches, in generating valuable information on the effects of, and responses to, environmental stressors upon biological systems (e.g., [91–94]). The availability and accessibility of numerous genomic techniques and their approaches have made them favorable tools that are growing in use within interdisciplinary research fields. High-throughput gene and protein techniques are currently encompassed into systems biology [90,95], attempting to highlight the complex interactions between responses that span from cellular mechanisms to the whole animal, and extrapolating these, via life cycle models to population level effects.

Genomic approaches, or “omics” as they are currently referred to, encompass transcriptomics (mRNA), proteomics (protein), and metabolomics (metabolic signatures from resulting activity), and incorporate epigenetics (heritable changes in expression), and genotyping (DNA). The proliferation of different genomic approaches over the past decade, through a combination of advances in biological, instrumental and bioinformatic techniques, has thus generated a means of characterizing biological processes in response to stressors, yielding unparalleled information on the molecular and biochemical status of organisms. Numerous reviews are currently available on the perspectives and future directions for the inclusion of mechanistic research in aquatic toxicology (e.g., [96]).

**Transcriptomics**, otherwise known as “global analysis of gene expression”, examines the genes and corresponding biochemical pathways that are involved in various biological processes. These techniques are highly sensitive indicators of an organisms' interaction with their environment, because they capture the physiological responses initiated at the molecular level. However, it is important to highlight that RNA is not the final product of a gene and that the functional expression of a gene is carried out by the synthesized protein. Protein expression, however, is influenced and regulated by many processes downstream of mRNA synthesis. Transcriptional expression levels can be utilized to understand the functional network of the biochemical pathways they are involved in, and thus are useful in the characterization of biological processes of interest. Notable examples are those of Spromberg and Meador [97], who were able to associate immune suppression, reproductive dysfunction and somatic growth impairment in salmonids, to population level responses using life history models, Heckmann *et al.* [98] and Connon *et al.* [99] who were successful in combining molecular and organismal stress responses in *Daphnia magna*, by assessing mechanistic responses to exposure indicative of chronic consequences for population growth rates, and Garcia-Reyero *et al.* [92,100] who investigated synergistic and antagonistic effect of EDC mixtures, identifying specific signatures relating to the modes of action of each chemical, and highlight the complexities of assessing multiple compound in wastewater treatment effluent. Studies of this kind emphasize the importance of bridging the assessment of toxicity responses at environmentally relevant (or low) concentrations that can mechanistically explain the effects contaminants may have at higher levels of biological organization and resulting population dynamics.

**Proteomics** represents the high-throughput assessment of the functional responses of gene expression; the proteins and peptides, as well as protein-protein interactions. Combined with bioinformatic analyses, proteomics is used to assess functional biochemical pathways that respond to environmental and/or contaminant stimuli. As indicated above, these responses may not necessarily correspond with transcriptional changes due to factors, including rates of degradation, translational inefficiencies, and relativity to the status of the cell at the assessed time [90]. As such they may offer a more robust approach for ecotoxicological risk assessments [93], however analysis is somewhat limited by the relatively large sample volume requirements. Proteomic tools have been successfully utilized in studies identifying hypoxic effects in fish brains [101], chemical and site-specific signatures [94,102], specific effects from exposures to microcystins [103,104], combinations of transcriptomics and proteomics in the assessment of neurological damage in response to dieldrin exposure [105], as well as in numerous studies investigating the effects of endocrine disrupting chemicals (as reviewed by Martyniuk and Denslow [106]).

**Metabolomics** measures the concentrations of metabolites that represent enzymatic activity upon xenobiotics, and associates these, through bioinformatic techniques, with changes in biological functions in the exposed organism, thus its development and continual enhancement has contributed significantly to the field of ecotoxicology. A major advantage that metabolomics has over transcriptomics and proteomics, is that resulting metabolites do not differ between taxa, thus this approach is not species-specific, permitting for the same toolbox to be used a broad range of species.

Lin *et al.* [107] summarize the emergence of metabolomics within the “omics” field and highlight how these methodologies can be used within regulatory ecotoxicology, and Ludwig and Viant [108] present a valuable review of advances in metabolomics methodologies that enhance the confidence in metabolite identification and reduce batch-to-batch variation. One of the main benefits of metabolomics is that it provides a highly suitable assessment approach for field studies. Prominent studies in metabolomics include those of Viant [109] who describes the use of metabolite profiles to determine Chinook salmon exposure responses, differentiating between amongst three classes of pesticides, Ekman *et al.* [110], who used metabolite analyses linking feminization of male fathead minnows on exposure to EE2, and Poynton *et al.* [111] who utilized a combination of transcriptomics and metabolomics on *D. magna* haemolymph, to assess effects of sublethal cadmium exposure, identifying decreased levels of digestive enzymes, elevated transcription of cuticular proteins, and genes involved in oxidative stress that corresponded with effects on amino acids and fatty acids determined via metabolomics. Recent advantages, capabilities and applications of metabolomics tools within ecological research fields ranging from ecophysiology to ecotoxicology are reviewed in Viant *et al.* [112].

Whilst these technologies have proven to be useful in elucidating modes of action of toxicants and can contribute to the risk assessment process as part of a weight-of-evidence approach, there is a strong need for critical studies that relate molecular changes to ecological adverse outcome [113], as well as for studies addressing mixture effects [114]. There are several examples, however, which suggest that mechanistic links between omics responses and effects at other levels of biological organization (behavior, growth, predation risk, fitness, and mortality) can be established (e.g., [115–118]). Omics technologies continue to advance, becoming more affordable, efficient and generating rich datasets, which is facilitating the incorporation of these techniques into risk assessments and assisting regulatory decisions [113].

**Epigenetics** is an emerging field that is rapidly being incorporated into ecotoxicological studies. It investigates the alterations in gene function or cell phenotype, without changes in DNA sequences, that may result, for example from methylation, or histone modification, and can be triggered by alterations in environmental stressors [119,120]. Vandegehuchte and Janssen [121] and Head *et al.* [120] review the effects of epigenetic induced changes in unexposed offspring, stressing that populations can experience the effects of their ancestors' exposure to chemicals, and that there is a need to also evaluate the persistence of chemical exposure-induced epigenetic effects in multiple subsequent generations. Harris *et al.* [122] described *Daphnia magna* as being an ideal model organism for epigenetic research as its parthenogenetic life-cycle permits assessments to be conducted in the absence of confounding genetic differences. Additional attributes that make *D. magna* an optimal and internationally recognized model organism for ecotoxicological studies are its rapid life-cycle, ease of culture and low cost, as well as the occurrence of multiple phenotypic differentiations, so-called polyphenisms, leading to modifications such as neckteeth and helmets in response to hormonal cues from predators (e.g., *Chaoborus* spp). Vandegehuchte *et al.* [123,124] evaluated DNA cytosine methylation transgenerational effects in populations of *D. magna*, which resulted in decreases in body length and reduced numbers of offspring following contaminant exposure.

Finally, another genomic approach that will undoubtedly be increasingly incorporated into ecotoxicological assessments is **genotyping**. It is potentially genotyping techniques that will assist comparative species toxicology, and help understand what sensitivity means from one organism to another. This is an especially important parameter that must be considered within the context of ecosystem resilience, as differences in intra-species sensitivity will affect both genetic structure of populations as well as community structure, especially in the presence of a multitude of contaminants within aquatic ecosystems. Morgan *et al.* [125] highlight the need for the awareness of microevolution in ecotoxicological studies, using high levels of genetically differentiated metal resistance in invertebrate populations as an example. This review advocates the inclusion of genotyping tools, particularly in the assessment of field populations, in order to understand and incorporate likely adaptations of sampled organisms to polluted sites. Babin-Fenske *et al.* [126] conducted a phylogenetic analysis of *Hyallela azteca* species, contrasting the different lake habitats, revealing two distinct populations associated with historically contaminated and low impacted lakes, respectively. Thus, when attempting to conduct comparisons using field-caught organisms, or for laboratory studies using sentinel organisms, it is essential to include genotype assessments in conjunction with epigenetic studies in order to understand relative intra- and inter-species sensitivities.

None of these techniques would be of use without bioinformatic approaches and databases used to store gained knowledge. The bioinformatic technology is continually attempting to assess the ever-increasing datasets, and has also expanded exponentially in recent years, but is somewhat restricted by the provision of limited data through existing databases (e.g., Toxnet, Comparative Toxicogenomics Database) on interactions between disease contaminant effects and adverse outcomes that are based on published data.

It is not the use of individual tools, determining individual endpoints, but rather an integration of many fields of expertise; multidisciplinary efforts, which will permit the generation of more accurate toxicological information to effectively guide ecotoxicology and environmental management in the future. Ecotoxicologists are especially aware of this, and thus are rapidly moving away from single endpoint assessments to investigating biological systems or pathway effects that may lead to adverse outcomes. Numerous reviews and studies are addressing this need for integration of many schools of thought. Kramer *et al.* [127] reviewed the application of a suite of techniques within ecotoxicology that will permit bridging of mechanistic approaches with population-level effects. Ankley *et al.* [128] report the use of a multidisciplinary approach in which mechanistic assessments, in conjunction with bioinformatics, modeling, and whole organism tests, successfully determine links across biological levels of organization. Not only are multidisciplinary approaches suitable for the assessment of contaminant exposure, but are also directly applicable to disease, which will directly affect sensitivity to contaminants and vice versa [129]. Hostetter *et al.* [130] and Connon *et al.* [131] describe the use of a multidisciplinary approach synchronously utilizing molecular indicators of health status, with histopathology, external parameters of disease, and infection classes, which were strong predictors of survival probability of out-migrating salmonids. Similarly, Miller *et al.* [132], assess similar responses using genome-wide studies that predict salmonid migration and spawning failure.

## 4. From Genes to Populations

In order to link the effects of pollutants through various levels of biological organization to ecosystem health, an integrated and multifaceted approach is required, that is based on existing knowledge that correlates processes that include the route of uptake, detoxification and pathology in between each of the assessed levels [133]. Portfolios of carefully selected biological indicators, including standard whole organism bioassays as well as biomarkers and *in vitro* tests, are required to evaluate toxicity. There is also a need to directly link chemical pollution in ecosystems to measurable toxic effects [134]. Chemical concentration data can only be of value for environmental impact assessments when effect levels and mechanisms of action of chemicals are already known. Thus the value of biological testing cannot be underestimated when attempting to detect toxicity before populations are negatively impacted. The sensitivity of an organism to a particular contaminant relates to its ability to mechanistically cope with the exposure; the ability of an organism to sequester or eliminate resulting metabolites from its body, and its overall health status. Recent mechanistic research approaches, such as the omics techniques described above, have permitted the assessment of sublethal toxicology, driving experimental designs to encompass environmentally relevant, low dose contaminant concentrations that are often below the limit of detection of current analytical chemistry protocols.

The majority of omics studies published within the last decade (too many to cite) have relied on more than the interpretation of genes, proteins or metabolite data, that indicate mechanistic effects, or modes of action of contaminants. Most of these studies have also integrated at least one other endpoint, at higher levels of biological organization; associating mechanistic responses with reproductive growth rate, offspring viability and sex ratios, masculinization or feminization in fish, disease, growth and development or survival, or with other physiological parameters such as swimming performance and mating behavior. The greater the integration of the above endpoints, the greater will be the need for multidisciplinary collaborations, and greater will be our understanding of the adverse effects caused by single contaminants, their mixtures, and their interactions with biotic and abiotic parameters within a particular ecosystem.

Adverse outcome pathways (AOPs) have been defined by Ankley *et al.* [135] as “*a conceptual construct that portrays existing knowledge concerning the linkage between a direct molecular initiating event (e.g., a molecular interaction between a xenobiotic and a specific biomolecule) and an adverse outcome at a biological level of organization relevant to risk assessment*”. This approach establishes a basis upon which concepts and testable hypotheses, to be tested by multi-disciplinary fields of study, can be constructed. Examples of such studies include those of Villeneuve *et al.* [136] who provide a review of the integration of toxicogenomics, as a complementary tool to traditional methodologies, when applied to risk assessment. Altenburger *et al.* [114] highlight the use of omics technology for mixture studies, stressing the need for dose and time mixture toxicity models that include bioinformatics and stress response concepts that can integrate the multiple responses into testable hypotheses. Testable hypotheses will permit the understanding and thus the prediction of an adverse effect. In order to adopt this approach, researchers need more than endpoints at the mechanistic and population levels within a study; assessments at associated and subsequent levels of biological organization are required.

**Figure 5.**
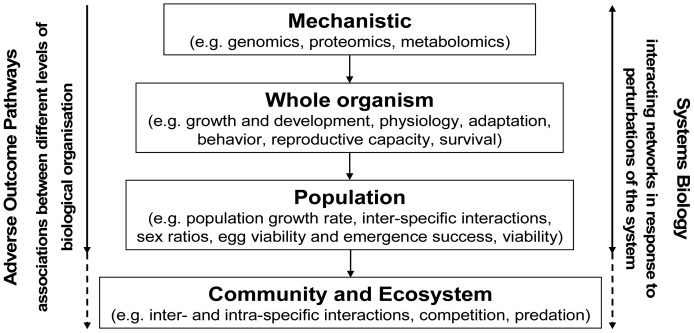
A suite of assessment types may be used to investigate links or associations across different levels of biological organization, as well as the interactions amongst networks in response to perturbations within a biological system.

As indicated above, biological systems do not respond to perturbations as single molecules, nor through single pathways, but rather as multiple-level interactions, within networks of cellular components [137]. While AOP assessments aim to establish associations between different levels of biological organization, systems biology aims at understanding impacts of contaminants upon the whole (Figure 5). Systems biology is thus defined as the assessment of interacting networks in response to perturbations in order to discover, understand and predict the emerging properties of the system [137], by examining the dynamics of cellular to organismal functions, rather than its individual components [138].

By far, the greatest benefit of systems biology is that it is based on conceptual, descriptive and mathematical models used to describe these perturbations, and their value in determining effective outcomes at higher level of organization. A recent example of such approach involves the use of reverse engineering; employing mathematical and statistical algorithms to determine relationships between genes, proteins and metabolites. Perkins *et al.* [139] describe this approach utilizing high-density transcription data to identify AOPs that relate to the disruption of the hypothalamus-pituitary-gonadal endocrine axis in fathead minnows. The authors then assessed this approach in contrast with biochemical biomarkers; vitellogenin, testosterone and estrogen, as surrogates of fecundity, inferring effects at higher levels of biological organization.

Current and planned studies based on AOP approaches are expected to strengthen the knowledge base required for systems biology to become a successful predictive tool in the ecotoxicological context, and for this information to be used towards understanding pollution effects on community and ecosystem functioning.

## 5. The Concepts of Ecosystem Functioning and Ecosystem Services in the Context of Ecotoxicology

One of the greatest challenges in ecotoxicology ultimately lies in linking the effects of chemical exposure to ecosystem-level effects and ecosystem services [140]. Generally, effects on ecosystem functioning and effects on ecosystem services need to be distinguished. Ecosystem effects comprise all abiotic and biotic changes that exceed the natural change rate or frequency. In some cases, such changes can strongly affect the functioning of entire ecosystems, so-called phase shifts. On the other hand, the concept of ecosystem services relies on all values and functions that ecosystems provide for mankind, e.g., including provision of food, recreation, aesthetic and ethical values [140,141]. Ecosystem services are particularly strongly valued if direct links between contamination and human health are evident, e.g., in the case of fishes that exceed certain levels of methylmercury and cannot be eaten or sold. From the perspective of ecosystem functioning, the interaction of chemical stressors with biodiversity is most important, comprising the range from genetic diversity, to species, communities, and habitat types. Whilst effects on higher levels of biological organization are more obvious, contaminant pollution also has the potential to exert subtle selective power on the gene pool, e.g., by selecting or favoring individuals that are more resistant to certain types of contaminants than others. Such selection can quickly (*i.e.*, within few generations) result in evolution of gene pools, e.g., the reduction of genetic variation.

Traditionally, most studies in the context of assessing ecosystem effects rely on time-series correlations between contaminant concentrations and monitored community-level changes in particular types of habitat. One major weakness of such an approach is that correlation-based analyses are not suitable for verifying cause and effect, that changes on an ecosystem-level often lack replication and are typically induced by many other factors (e.g., temperature or salinity shifts) that cannot be separated from the effects of contaminants. At the same time, such approaches require a high monitoring effort in the field and, with few exceptions (e.g., [27]), contamination of natural ecosystems for scientific experiments are not considered ethical. Consequently, ecosystem level effects are typically being studied in small-scale mesocosms or microcosms that resemble natural ecosystems, containing at least a minimum number of representative species, ideally also keystone species whose presence or absence critically influences the entire functioning of such systems. Mesocosms have been widely applied to identify long-term community effects of low toxicant concentrations but often face the problem that results are obscured by confounding factors and high variability between replicates (e.g., [142,143]). This variability has been identified as a major source of false negative results, *i.e.*, concluding a chemical has no effect when it really has [143–146]. Whether community responses can better be evaluated using a species-based principle response curve (PRC) approach [147] or with the SPEAR_mesocosm_ approach [142,148] is still controversial [149,150].

In cases in which no data on ecosystem effects are available, modeling approaches are typically being applied. Traditionally, these approaches derive “safe” environmental concentrations of toxicants by extrapolation factors or by statistical extrapolation from a set of single species toxicity data. One major weakness of such approaches is that species' interactions which can be important in natural ecosystems (e.g., predator-prey interactions) are not considered. Ecology-based ecological modeling alternatives have been suggested to account for ecological interactions, but it is largely unexplored how well predictions from these models quantitatively match with large-scale experimental studies [151].

## 6. Conclusions and Outlook

Chemical characterization by itself does not provide specific biological information about potential hazards to organisms. Traditional ecotoxicological approaches that utilize individual endpoints lead to assumptions and uncertainty in factors that can erroneously infer effects at higher levels of biological organization. By truly integrating monitoring strategies, involving chemical analyses, a suite of ecotoxicological tools, the study of population/community responses in the same water body and, preferably, time of the year, a more holistic picture can be obtained. Such an approach can tell us whether the chemicals analyzed are bioavailable and giving rise to negative health effects in aquatic organisms, and whether population effects are observed. The concept of weight of evidence (WOE) refers to the integration of data generated within such a multidisciplinary approach including data from different studies, or lines of evidence (LOEs). Thus in the 21st century, ecotoxicology needs to evolve, moving away from traditional toxicology approaches that are still in use by most regulatory agencies, and encompass integrative tools and models to evaluate the risk posed by existing and new contaminants as part of their registration, potential use, and application. Ideally, integrative approaches of linking mechanistic responses on the cellular level with whole organism, population, community and ecosystem effects and services would be desirable for a comprehensive assessment of ecotoxicological risk. Since such approaches are difficult and costly, the identification of key links between responses at different levels of biological organization with ecosystem functioning, and associations with ecosystem services, can be a first useful step towards making ecotoxicology more fundamental and effective.

## Figures and Tables

**Table 1.** Assessment or safety factors for the derivation of environmental quality standards from toxicity data [12].

**Available data**	**Assessment/safety factor**
At least one short-term LC_50_/EC_50_ from each of three trophic levels (fish, invertebrates (preferred *Daphnia*), and algae)	1,000
One long-term EC_10_ or NOEC (either fish or *Daphnia*)	100
Two long-term results from (e.g., EC_10_ or NOECs) from species representing two trophic levels (Fish and/or *Daphnia* and/or algae)	50
Long-term results (e.g., EC_10_ or NOECs) from at least three species (normally fish, *Daphnia* and algae) representing three trophic levels	10
Species sensitivity distribution (SSD) method	5-1 (to be justified case-by-case)
Field data or model ecosystem	Reviewed on a case-by-case basis

**Table 2.** Examples of promising effect-based *in vitro* tests and chemicals they respond to (ISO: International Organization for Standardization; DIN: Deutsches Institut für Normung; German Institute for Standardization).

**Effect**	***In vitro* Test**	**Responsive to**	**Standard Protocol/Reference**
Aryl hydrocarbon (Ah) receptor (in-) activation	Recombinant cell based assays, e.g., H4IIE-luc, AhR-CAFLUX, DR-CALUX^®^	Dioxins, coplanar polychlorinated biphenyls (PCBs), poly-aromatic hydrocarbons (PAHs)	[21] BioDetection Systems BV, Amsterdam, NL
			[28] (US EPA, Method 4435, 2007)
			[29]
(Anti-) Estrogenicity	(anti-) Yeast estrogen screen (YES)	Natural and synthetic estrogens, bisphenol A, nonylphenol, phthalates (anti), and others.	[23]
	Cell-based reporter gene assays, such as T47D. Luc, T47D-KBluc, ER CALUX^®^		[30]
			[31]
			[21] BioDetection Systems BV, Amsterdam, NL
	Cell proliferation assay (MCF-7, E-screen)		[32]
(Anti-) Androgenicity	(anti-) yeast androgen screen (YAS)	Natural and synthetic androgens, e.g., androstanedione	[33]
	Cell-based reporter gene assays, such as AR-CALUX^®^		[21] BioDetection Systems BV, Amsterdam, NL
Thyroid hormone disruption	Transthyretin (TTR)-binding assay	Polybrominated diphenyl ethers (PBDEs), PCBs, and other halogenated phenols, pesticides	[34]
	Cell-based reporter gene assays, such as TR CALUX^®^		[21] BioDetection Systems BV, Amsterdam, NL
	Cell proliferation assay (T-Screen)		[35]
Genotoxicity/DNA damage	Ames assay	Heavy metals, pesticides, PAHs and others	ISO 16240, 2005, DIN 38415-3, 1999
	UmuC-assay		ISO13829, 2000
	Micronucleus assay		ISO 21427-2, 2006
Neurotoxicity	Inhibition of acetylcholinesterase	Organophosphate/carbamate insecticides	DIN 38415-1
			[36]
Inhibition of Photosynthesis	Combined algae test	Herbicides	[37]
Cytotoxicity	Microtox (luminescent bacteria) assay	Unspecific	ISO 11348-3
			[37]
	Cell viability assays such as MTT or neutral red staining	Unspecific	[38]
			[39]

**Table 3.** Species, exposure periods, test types and endpoints used for some standardized *in vivo* bioassays in freshwater.

**Organism**	**Exposure period**	**Test type**	**Toxicity Endpoint**	**Standard protocols**
Green algae:				
*Pseudokirchneriella subcapitata*	72–96 h	chronic	growth	ISO 8692, [40]
Waterflea:				
*Daphnia magna*	48 h	acute	mortality	ISO 6341-L40,
	21 days	chronic	fecundity	US EPA, 2002
*Ceriodaphnia dubia*	96 h	acute	mortality	ISO 20665, [40]
	7–8 days	chronic	fecundity	
Fish:				
*Danio rerio*	96 h	acute	mortality	ISO 7346
*Oncorhynchus mykiss*	28 days	chronic	growth	ISO 10229
*Pimephales promelas*	96 h	acute	mortality	[40,41]
	7 days	chronic	growth	

**Table 4.** Examples of promising biomarkers used to assess exposure to chemicals, and health status of wild fish in environmental monitoring.

**Effect**	**Biomarker**	**Standard Protocol/Reference**
Ah receptor	CYP1A (mRNA, protein)	[58,64]
(in-) activation	EROD-Assay	ISO/TS 23893-2
Metal sequestration	Metallothionein (protein)	[59]
Endocrine disruption	Vitellogenin, protein	ISO/DIS 23893-3
	Vitellogenin, mRNA	(under development), [65]
	CYP19/aromatase (mRNA, protein, activity)	[66]
	Steroid levels (blood)	[67]
	Histopathological changes in gonadal tissue	[68]
Thyroid hormone disruption	Transthyretin (TTR), mRNA	[69]
	Thyroid hormone levels	
Genotoxic effects	Micronucleus test	ISO 12427-2
	DNA adducts	[70]
	DNA strand breaks (Comet assay)	[71]
Oxidative stress	Lipid peroxidation	[58]
	Phase II enzymes and cofactors	[62,72]
	Antioxidant enzymes	
Neurotoxicity	Inhibition of acetylcholinesterase activity	[73,74]
Immunotoxicity	Phagocytic activity	[75]
	Macrophage aggregates	[76]
	Other blood parameters	[77]
Cellular integrity	Neutral red retention assay (lysosomal stability assay)	[78]
Cell/Tissue damage	Histopathological changes	[60]

## References

[1] Diamond J.M. (2005). Collapse: How Societies Choose to Fail or Succeed.

[2] Hickey J.J., Anderson D.W. (1968). Chlorinated hydrocarbons and eggshell changes in raptorial and fish-eating birds. Science.

[3] Vos J.G., Dybing E., Greim H.A., Ladefoged O., Lambre C., Tarazona J.V., Brandt I., Vethaak A.D. (2000). Health effects of endocrine-disrupting chemicals on wildlife, with special reference to the European situation. Crit. Rev. Toxicol..

[4] Baumann P.C., Harshbarger J.C. (1995). Decline in liver neoplasms in wild brown bullhead catfish after coking plant closes and environmental PAHs plummet. Environ. Health Persp..

[5] Depledge M.H., Galloway T.S. (2005). Healthy animals, healthy ecosystems. Front. Ecol. Environ..

[6] CAS Homepage of the Chemical Abstracts Service. http://www.cas.org.

[7] Werner I., Geist J., Okihiro M., Rosenkranz P., Hinton D.E. (2002). Effects of dietary exposure to the pyrethroid pesticide esfenvalerate on medaka (*Oryzias latipes*). Mar. Environ. Res..

[8] Hamers T., Krogh P.H. (1997). Predator and prey relationships in a two-species toxicity test system. Ecotoxicol. Environ. Saf..

[9] Hooper H.L., Sibly R.M., Hutchinson T.H., Maund S.J. (2003). The influence of larval density, food availability and habitat longevity on the life history and population growth rate of the midge Chironomus riparius. Oikos.

[10] Vandenberg L.N., Colborn T., Hayes T.B., Heindel J.J., Jacobs D.R., Lee D.-H., Shioda T., Soto A.M., vom Saal F.S., Welshons W.V. (2012). Hormones and endocrine-disrupting chemicals: Low-dose effects and nonmonotonic dose responses. Endocr. Rev..

[11] European Commission Working Document, Guidance Document on Aquatic Ecotoxicology in the context of the Directive 91/414/EEC (Sanco/3268/2001 rev.4 (final). http://ec.europa.eu/food/plant/protection/evaluation/guidance/wrkdoc10_en.pdf.

[12] European Commission Common Implementation Strategy for the Water Framework Directive (2000/602/EC). http://ec.europa.eu/environment/water/water-framework/objectives/pdf/strategy3.pdf.

[13] European Union Directive 2000/60/EC of the European Parliament and of the Council of 23 October 2000 Establishing a Framework for Community Action in the Field of Water Policy. http://eur-lex.europa.eu/LexUriServ/LexUriServ.do?uri=CELEX:32000L0060:en:HTML.

[14] Crane M., Babut M. (2007). Environmental quality standards for water framework directive priority substances: Challenges and opportunities. Integrated Environ. Assess. Manag..

[15] Gilliom R.J., Barbash J.E., Kolpin D.W. (1999). Testing water quality for pesticide pollution. Environ. Sci. Technol..

[16] Schwarzenbach R.P., Escher B.I., Fenner K., Hofstetter T.B., Johnson C.A., von Gunten U., Wehrli B. (2006). The challenge of micropollutants in aquatic systems. Science.

[17] Backhaus T., Sumpter J., Blanck H., Kümmerer K. (2008). On the Ecotoxicology of Pharmaceutical Mixtures Pharmaceuticals in the Environment.

[18] Meek M.E., Boobis A.R., Crofton K.M., Heinemeyer G., van Raaij M., Vivkers C. (2011). Risk assessment of combined exposure to multiple chemicals: A WHO/IPCS framework. Regul. Toxicol. Pharmacol..

[19] Backhaus T., Faust M. (2012). Predictive environmental risk assessment of chemical mixtures: A conceptual framework. Environ. Sci. Technol..

[20] Escher B., Leusch F. (2012). Bioanalytical Tools in Water Quality Assessment.

[21] Van der Linden S.C., Heringa M.B., Man H.-Y., Sonneveld E., Puijker L.M., Brouwer A., van der Burg B. (2008). Detection of multiple hormonal activities in wastewater effluents and surface water, using a panel of steroid receptor CALUX bioassays. Environ. Sci. Technol..

[22] Leusch F.D.L., de Jager C., Levi Y., Lim R., Puijker L., Sacher F., Tremblay L.A., Wilson V.S., Chapman H.F. (2010). Comparison of five *in vitro* bioassays to measure estrogenic activity in environmental waters. Environ. Sci. Technol..

[23] Routledge E.J., Sumpter J.P. (1996). Estrogenic activity of surfactants and some of their degradation products assessed using a recombinant yeast screen. Environ. Toxicol. Chem..

[24] Sumpter J.P. (2005). Endocrine disrupters in the aquatic environment: An overview. Acta Hydrochim. Hydrobiol..

[25] Martinović D., Hogarth W.T., Jones R.E., Sorensen P.W. (2007). Environmental estrogens suppress hormones, behavior, and reproductive fitness in male fathead minnows. Environ. Toxicol. Chem..

[26] Johnson A.C., Williams R.J. (2004). A model to estimate influent and effluent concentrations of estradiol, estrone, and ethinylestradiol at sewage treatment works. Environ. Sci. Technol..

[27] Kidd K.A., Blanchfield P.J., Mills K.H., Palace V.P., Evans R.E., Lazorchak J.M., Flick R.W. (2007). Collapse of a fish population after exposure to a synthetic estrogen. Proc. Natl. Acad. Sci. USA.

[28] Nagy S.R., Sanborn J.R., Hammock B.D., Denison M.S. (2002). Development of a green fluorescent protein-based cell bioassay for the rapid and inexpensive detection and characterization of a receptor agonists. Toxicol. Sci..

[29] Horii Y., Khim J.S., Higley E.B., Giesy J.P., Ohura T., Kannan K. (2009). Relative potencies of individual chlorinated and brominated polycyclic aromatic hydrocarbons for induction of aryl hydrocarbon receptor-mediated responses. Environ. Sci. Technol..

[30] Legler J., van den Brink C.E., Brouwer A., Murk A.J., van der Saag P.T., Vethaak A.D., van der Burg B. (1999). Development of a stably transfected estrogen receptor-mediated luciferase reporter gene assay in the human T47D breast cancer cell line. Toxicol. Sci..

[31] Wilson V.S., Bobseine K., Gray L.E. (2004). Development and characterization of a cell line that stably expresses an estrogen-responsive luciferase reporter for the detection of estrogen receptor agonist and antagonists. Toxicol. Sci..

[32] Soto A.M., Sonnenschein C., Chung K.L., Fernandez M.F., Olea N., Serrano F.O. (1995). The E-SCREEN assay as a tool to identify estrogens: An update on estrogenic environmental pollutants. Environ. Health Perspect.

[33] Sohoni P., Sumpter J.P. (1998). Several environmental oestrogens are also anti-androgens. J. Endocrinol.

[34] Weiss J.M., Andersson P.L., Lamoree M.H., Leonards P.E.G., van Leeuwen S.P.J., Hamers T. (2009). Competitive binding of poly- and perfluorinated compounds to the thyroid hormone transport protein transthyretin. Toxicol. Sci..

[35] Gutleb A.C., Meerts I.A.T.M., Bergsma J.H., Schriks M., Murk A.J. (2005). T-Screen as a tool to identify thyroid hormone receptor active compounds. Environ. Toxicol. Pharmacol..

[36] Hamers T., Molin K.R.J., Koeman J.H., Murk A.J. (2000). A small-volume bioassay for quantification of the esterase inhibiting potency of mixtures of organophosphate and carbamate insecticides in rainwater: Development and optimization. Toxicol. Sci..

[37] Escher B.I., Bramaz N., Mueller J.F., Quayle P., Rutishauser S., Vermeirssen E.L.M. (2008). Toxic equivalent concentrations (TEQs) for baseline toxicity and specific modes of action as a tool to improve interpretation of ecotoxicity testing of environmental samples. J. Environ. Mon..

[38] Mosmann T. (1983). Rapid colorimetric assay for cellular growth and survival: application to proliferation and cytotoxicity assays. J. Immunol. Meth..

[39] Borenfreund E., Puerner J.A. (1985). Toxicity determined *in vitro* by morphological alterations and neutral red absorption. Toxicol. Lett..

[40] (2002). Short-Term Methods for Measuring the Chronic Toxicity of Effluents and Receiving Waters to Freshwater Organisms.

[41] (2002). Methods for Measuring the Acute Toxicity of Effluents and Receiving Waters to Freshwater and Marine Organisms.

[42] (2006). United Nations Globally Harmonized System of Classification and Labelling of Chemicals (GHS).

[43] Delignette-Muller M.-L., Forfait C., Billoir E., Charles S. (2011). A new perspective on the Dunnett procedure: Filling the gap between NOEC/LOEC and ECx concepts. Environ. Toxicol. Chemi..

[44] (1991). Methods for Aquatic Toxicity Identification Evaluations: Phase I, EPA/600/6-91/003.

[45] (1993). Methods for Aquatic Toxicity Identification Evaluations: Phase II, EPA/600/R-92/080.

[46] Werner I., Deanovic L.A., Connor V., de Vlaming V., Bailey H.C., Hinton D.E. (2000). Insecticide-caused toxicity to *Ceriodaphnia dubia* (CLADOCERA) in the Sacramento-San Joaquin River delta, California, USA. Environ. Toxicol. Chem..

[47] Brack W. (2003). Effect-directed analysis: A promising tool for the identification of organic toxicants in complex mixtures?. Anal. Bioanal. Chem..

[48] Bandow N., Altenburger R., Streck G., Brack W. (2009). Effect-directed analysis of contaminated sediments with partition-based dosing using green algae cell multiplication inhibition. Environ. Sci. Technol..

[49] OECD Homepage of Organization for Economic Co-operation and Development. http://www.oecd.org.

[50] Werner I., Deanovic L.A., Miller J., Denton D.L., Crane D., Mekebri A., Moore M.T., Wrysinski J. (2010). Use of vegetated agricultural drainage ditches to decrease toxicity of irrigation runoff from tomato and alfalfa fields in California, USA. Environ. Toxicol. Chem..

[51] Burton G.A., Greenberg M.S., Rowland C.D., Irvine C.A., Lavoie D.R., Brooker J.A., Moore L., Raymer D.F.N., McWilliam R.A. (2005). *In situ* exposures using caged organisms: A multi-compartment approach to detect aquatic toxicity and bioaccumulation. Environ. Pollut..

[52] Triebskorn R., Adam S., Behrens A., Beier S., Böhmer J.R., Braunbeck T., Casper H., Dietze U., Gernhöfer M., Honnen W., Köhler H.-R. (2003). Establishing Causality between pollution and effects at different levels of biological organization: The VALIMAR project. Hum. Ecol. Risk Assess.

[53] Lopes I., Baird D.J., Ribeiro R. (2005). Resistance to metal contamination by historically-stressed populations of Ceriodaphnia pulchella: Environmental influence *versus* genetic determination. Chemosphere.

[54] Baird D.J., Burton G.A., Culp J.M., Maltby L. (2007). Summary and recommendations from a SETAC pellston workshop on *in situ* measures of ecological effects. Integrated Environ. Assess. Manag..

[55] Baird D.J., Brown S.S., Lagadic L., Liess M., Maltby L., Moreira-Santos M., Schulz R., Scott G.I. (2007). *In situ*-based effects measures: Determining the ecological relevance of measured responses. Integrated Environ. Assess. Manag..

[56] Wharfe J., Adams W., Apitz S.E., Barra R., Bridges T.S., Hickey C., Ireland S. (2007). *In situ* methods of measurement—An important line of evidence in the environmental risk framework. Integrated Environ. Assess. Manag..

[57] Huggett R.J., Kimerly R.A., Mehrle P.M., Bergman H.L. (1992). Biomarkers: Biochemical, Physiological and Histological Markers of Anthropogenic Stress.

[58] Stegeman J.J., Brouwer M., Richard T.D.G., Foerlin L., Fowler B.A., Sanders B.M., van Veld P.A., Huggett R.J., Kimerly R.A., Mehrle P.M., Bergman H.L. (1992). Molecular responses to environmental contamination: Enzyme and protein systems as indicators of chemical exposure and effect. Biomarkers: Biochemical, Physiological and Histological Markers of Anthropogenic Stress.

[59] Roesijadi G., Robinson W.E., Malins D.C., Ostrander G.K. (1994). Metal regulation in aquatic animals: mechanisms of uptake, accumulation and release. Aquatic Toxicology, Molecular, Biochemical and Cellular Perspectives.

[60] Hinton D.E., Baumann P.C., Gardner G.C., Hawkins W.E., Hendricks J.D., Murchelano R.A., Okihiro M.S., Huggett R.J., Kimerly R.A., Mehrle P.M., Bergman H.L. (1992). Histopathologic biomarkers. Biomarkers: Biochemical, Physiological and Histological markers of Anthropogenic Stress.

[61] Forbes V.E., Palmqvist A., Bach L. (2006). The use and misuse of biomarkers in ecotoxicology. Environ. Toxicol. Chem..

[62] Van der Oost R., Beyer J., Vermeulen N.P.E. (2003). Fish bioaccumulation and biomarkers in environmental risk assessment: A review. Environ. Toxicol. Pharmacol..

[63] Dagnino A., Allen J.I., Moore M.N., Broeg K., Canesi L., Viarengo A. (2007). Development of an expert system for the integration of biomarker responses in mussels into an animal health index. Biomarkers.

[64] Stagg R.M., Rusin J., McPhail M.E., McIntosh A.D., Moffat C.F., Craft J.A. (2000). Effects of polycyclic aromatic hydrocarbons on expression of cyp1a in salmon (*Salmo salar*) following experimental exposure and after the *Braer* oil spill. Environ. Toxicol. Chemi..

[65] Sumpter J.P., Jobling S. (1995). Vitellogenesis as a biomarker for estrogenic contamination of the aquatic environment. Environ. Health Perspect.

[66] Rotchell J.M., Ostrander G.K. (2003). Molecular markers of endocrine disruption in aquatic organisms. J. Toxicol. Environ. Health B Crit. Rev..

[67] Aufartova J., Mahugo-Santana C., Sosa-Ferrera Z., Santana-Rodriguez J.J., Novakova L., Solich P. (2011). Determination of steroid hormones in biological and environmental samples using green microextraction techniques: An overview. Anal. Chim. Acta..

[68] Jobling S., Nolan M., Tyler C., Brighty G., Sumpter J.P. (1998). Widespread sexual disruption in wild fish. Environ. Sci. Technol..

[69] Rempel M.A., Schlenk D., Kwang W.J. (2008). Effects of Environmental estrogens and antiandrogens on endocrine function, gene regulation, and health in fish. International Review of Cell and Molecular Biology.

[70] Pfau W. (1997). DNA adducts in marine and freshwater fish as biomarkers of environmental contamination. Biomarkers.

[71] Singh N.P., McCoy M.T., Tice R.R., Schneider E.L. (1988). A simple technique for quantitation of low levels of DNA damage in individual cells. Exp. Cell Res..

[72] Winston G.W., di Giulio R.T. (1991). Prooxidant and antioxidant mechanisms in aquatic organisms. Aquat. Toxicol..

[73] Ellman G.L., Courtney K.D., Andres V., Feather-Stone R.M. (1961). A new and rapid colorimetric determination of acetylcholinesterase activity. Biochem. Pharmacol.

[74] Wheelock C.E., Eder K.J., Werner I., Huang H., Jones P.D., Brammell B.F., Elskus A.A., Hammock B.D. (2005). Individual variability in esterase activity and CYP1A levels in Chinook salmon (*Oncorhynchus tshawytscha*) exposed to esfenvalerate and chlorpyrifos. Aquat. Toxicol..

[75] Weeks B.A., Anderson D.P., DuFour A.P., Fairbrother A., Goven A.J., Lahvis G.P., Peters G., Huggett R.J., Kimerly R.A., Mehrle P.M., Bergman H.L. (1992). Immunologiocal biomarkers to assess environmental stress. Biomarkers: Biochemical, Physiological and Histological Markers of Anthropogenic Stress.

[76] Broeg K., Steinhagen D., Koehler A. (2008). Enzyme activities of liver macrophage aggregates as markers for immunotoxicity and Myxobolus sp infection in feral mullets (*Lisa aurata*). Mar. Environ. Res..

[77] Wester P.W., Vethaak A.D., van Muiswinkel W.B. (1994). Fish as biomarkers in immunotoxicology. Toxicology.

[78] Lowe D.M., Fossato V.U., Depledge M.H. (1995). Contaminant-induced lysosomal membrane damage in blood cells of mussels *Mytilus galloprovincialis* from the Venice Lagoon: An *in vitro* study. Mar. Ecol..

[79] Karr J.R. (1991). Biological integrity: A long-neglected aspect of water resource management. Ecol. Appl..

[80] Barbour M.T., Gerritsen J., Snyder B.D., Stribling J.D. (1999). Rapid Bioassessment Protocols for Use in Streams and Wadeable River: Periphyton, Benthic Macroinvertebrates and Fish.

[81] Friedrich G.E. (1990). Revision des Saprobiensystems. Z. Wasser. Abwass. Forsch..

[82] Liess M., SchÃfer R.B., Schriever C.A. (2008). The footprint of pesticide stress in communities: Species traits reveal community effects of toxicants. Sci. Total Environ..

[83] Beketov M.A., Foit K., Schäfer R.B., Schriever C.A., Sacchi A., Capri E., Biggs J., Wells C., Liess M. (2009). SPEAR indicates pesticide effects in streams: Comparative use of species- and family-level biomonitoring data. Environ. Pollut..

[84] Schafer R.B., von der Ohe P.C., Rasmussen J., Kefford B.J., Beketov M.A., Schulz R., Liess M. (2012). Thresholds for the effects of pesticides on invertebrate communities and leaf breakdown in stream ecosystems. Environ. Sci. Technol..

[85] Whitton B., Rott E., Friedrich G. Use of Algae for Monitoring Rivers.

[86] Araujo C.V.M., Blasco J., Moreno-Garrido I. (2010). Microphytobenthos in ecotoxicology: A review of the use of marine benthic diatoms in bioassays. Environ. Int..

[87] Blanck H., Wängberg S.-Ã. (1988). Induced community tolerance in marine periphyton established under arsenate stress. Can. J. Fish. Aquat. Sci..

[88] Blanck H. (2002). A critical review of procedures and approaches used for assessing Pollution-Induced Community Tolerance (PICT) in Biotic Communities. Hum. Ecol. Risk Assess.

[89] Truhaut R. (1977). Ecotoxicology: Objectives, principles and perspectives. Ecotoxicol. Environ. Saf..

[90] Garcia-Reyero N., Perkins E.J. (2011). Systems biology: Leading the revolution in ecotoxicology. Environ. Toxicol. Chem..

[91] Garcia-Reyero N., Adelman I.R., Martinovic D., Liu L., Denslow N.D. (2009). Site-specific impacts on gene expression and behavior in fathead minnows (Pimephales promelas) exposed *in situ* to streams adjacent to sewage treatment plants. BMC Bioinf..

[92] Garcia-Reyero N., Lavelle C.M., Escalon B.L., Martinovic D., Kroll K.J., Sorensen P.W., Denslow N.D. (2011). Behavioral and genomic impacts of a wastewater effluent on the fathead minnow. Aquat. Toxicol..

[93] Campos A., Tedesco S., Vasconcelos V., Cristobal S. (2012). Proteomic research in bivalves: Towards the identification of molecular markers of aquatic pollution. J. Proteomics.

[94] Thompson E.L., Taylor D.A., Nair S.V., Birch G., Hose G.C., Raftos D.A. (2012). Proteomic analysis of Sydney Rock oysters (*Saccostrea glomerata*) exposed to metal contamination in the field. Environ. Pollut..

[95] Ankley G.T., Miracle A., Perkins E.J., Daston G.P. (2008). Genomics in Regulatory Ecotoxicology: Applications and Challenges.

[96] Hahn M.E. (2011). Mechanistic research in aquatic toxicology: perspectives and future directions. Aquat. Toxicol..

[97] Spromberg J.A., Meador J.P. (2005). Relating results of chronic toxicity responses to population-level effects: Modeling effects on wild chinook salmon populations. Integrated Environ. Assess. Manag..

[98] Heckmann L.H., Sibly R.M., Connon R., Hooper H.L., Hutchinson T.H., Maund S.J., Hill C.J., Bouetard A., Callaghan A. (2008). Systems biology meets stress ecology: Linking molecular and organismal stress responses in Daphnia magna. Genome Biol..

[99] Connon R., Hooper H.L., Sibly R.M., Lim F.L., Heckmann L.H., Moore D.J., Watanabe H., Soetaert A., Cook K., Maund S.J. (2008). Linking molecular and population stress responses in Daphnia magna exposed to cadmium. Environ. Sci. Technol..

[100] Garcia-Reyero N., Kroll K., Liu L., Orlando E., Watanabe K., Sepulveda M., Villeneuve D., Perkins E., Ankley G., Denslow N. (2009). Gene expression responses in male fathead minnows exposed to binary mixtures of an estrogen and antiestrogen. BMC Genomics.

[101] Oehlers L.P., Perez A.N., Walter R.B. (2007). Detection of hypoxia-related proteins in medaka (Oryzias latipes) brain tissue by difference gel electrophoresis and de novo sequencing of 4-sulfophenyl isothiocyanate-derivatized peptides by matrix-assisted laser desorption/ionization time-of-flight mass spectrometry. Comp. Biochem. Physiol. C Toxicol. Pharmacol.

[102] Biales A.D., Bencic D.C., Flick R.L., Blocksom K.A., Lazorchak J.M., Lattier D.L. (2011). Proteomic analysis of a model fish species exposed to individual pesticides and a binary mixture. Aquat. Toxicol..

[103] Malecot M., Mezhoud K., Marie A., Praseuth D., Puiseux-Dao S., Edery M. (2009). Proteomic study of the effects of microcystin-LR on organelle and membrane proteins in medaka fish liver. Aquat. Toxicol..

[104] Wang M., Chan L.L., Si M., Hong H., Wang D. (2010). Proteomic analysis of hepatic tissue of zebrafish (Danio rerio) experimentally exposed to chronic microcystin-LR. Toxicol. Sci..

[105] Martyniuk C.J., Kroll K.J., Doperalski N.J., Barber D.S., Denslow N.D. (2010). Genomic and proteomic responses to environmentally relevant exposures to dieldrin: Indicators of neurodegeneration?. Toxicol. Sci..

[106] Martyniuk C.J., Denslow N.D. (2012). Exploring androgen-regulated pathways in teleost fish using transcriptomics and proteomics. Integr. Comp. Biol..

[107] Lin C.Y., Viant M.R., Tjeerdema R.S. (2006). Metabolomics: Methodologies and applications in the environmental sciences. J. Pestic. Sci..

[108] Ludwig C., Viant M.R. (2010). Two-dimensional J-resolved NMR spectroscopy: Review of a key methodology in the metabolomics toolbox. Phytochem. Anal..

[109] Viant M.R., Pincetich C.A., Tjeerdema R.S. (2006). Metabolic effects of dinoseb, diazinon and esfenvalerate in eyed eggs and alevins of Chinook salmon (*Oncorhynchus tshawytscha*) determined by ^1^H NMR metabolomics. Aquat. Toxicol..

[110] Ekman D.R., Teng Q., Villeneuve D.L., Kahl M.D., Jensen K.M., Durhan E.J., Ankley G.T., Collette T.W. (2008). Investigating compensation and recovery of fathead minnow (Pimephales promelas) exposed to 17α-ethynylestradiol with metabolite profiling. Environ. Sci. Tech..

[111] Poynton H.C., Taylor N.S., Hicks J., Colson K., Chan S., Clark C., Scanlan L., Loguinov A.V., Vulpe C., Viant M.R. (2011). Metabolomics of microliter hemolymph samples enables an improved understanding of the combined metabolic and transcriptional responses of Daphnia magna to Cadmium. Environ. Sci. Technol..

[112] Viant M., Sommer U. (2012). Mass spectrometry based environmental metabolomics: A primer and review. Metabolomics.

[113] Van Aggelen G., Ankley G.T., Baldwin W.S., Bearden D.W., Benson W.H., Chipman J.K., Collette T.W., Craft J.A., Denslow N.D., Embry M.R. (2010). Integrating omic technologies into aquatic ecological risk assessment and environmental monitoring: Hurdles, achievements, and future outlook. Environ. Health Perspect.

[114] Altenburger R., Scholz S., Schmitt-Jansen M., Busch W., Escher B.I. (2012). Mixture toxicity revisited from a toxicogenomic perspective. Environ. Sci. Technol..

[115] Geist J., Werner I., Eder K.J., Leutenegger C.M. (2007). Comparisons of tissue-specific transcription of stress response genes with whole animal endpoints of adverse effect in striped bass (*Morone saxatilis*) following treatment with copper and esfenvalerate. Aquat. Toxicol..

[116] Floyd E.Y., Geist J.P., Werner I. (2008). Acute, sublethal exposure to a pyrethroid insecticide alters behavior, growth, and predation risk in larvae of the fathead minnow (*Pimephales promelas*). Environ. Toxicol. Chem..

[117] Connon R.E., Geist J., Pfeiff J., Loguinov A.V., D'Abronzo L., Wintz H., Vulpe C.D., Werner I. (2009). Linking mechanistic and behavioral responses to sublethal esfenvalerate exposure in the endangered delta smelt; Hypomesus transpacificus (*Fam. Osmeridae*). BMC Genomics.

[118] Beggel S., Connon R., Werner I., Geist J. (2011). Changes in gene transcription and whole organism responses in larval fathead minnow (*Pimephales promelas*) following short-term exposure to the synthetic pyrethroid bifenthrin. Aquat. Toxicol..

[119] Legler J. (2010). Epigenetics: An emerging field in environmental toxicology. Integrated Environ. Assess. Manag..

[120] Head J.A., Dolinoy D.C., Basu N. (2012). Epigenetics for ecotoxicologists. Environ. Toxicol. Chem..

[121] Vandegehuchte M., Janssen C. (2011). Epigenetics and its implications for ecotoxicology. Ecotoxicology.

[122] Harris K.D.M., Bartlett N.J., Lloyd V.K. (2012). Daphnia as an emerging epigenetic model organism. Genet. Res. Int..

[123] Vandegehuchte M.B., Kyndt T., Vanholme B., Haegeman A., Gheysen G., Janssen C.R. (2009). Occurrence of DNA methylation in Daphnia magna and influence of multigeneration Cd exposure. Environ. Int..

[124] Vandegehuchte M.B., Lemière F., Vanhaecke L., Vanden Berghe W., Janssen C.R. (2010). Direct and transgenerational impact on *Daphnia magna* of chemicals with a known effect on DNA methylation. Comp. Biochem. Physiol. C Toxicol. Pharmacol.

[125] Morgan A.J., Kille P., Stürzenbaum S.R. (2007). Microevolution and ecotoxicology of metals in invertebrates. Environ. Sci. Technol.

[126] Babin-Fenske J.J., Merritt T.J.S., Gunn J.M., Walsh T., Lesbarrères D. (2012). Phylogenetic analysis of Hyalella colonization in lakes recovering from acidification and metal contamination. Can. J. Zool..

[127] Kramer V.J., Etterson M.A., Hecker M., Murphy C.A., Roesijadi G., Spade D.J., Spromberg J.A., Wang M., Ankley G.T. (2011). Adverse outcome pathways and ecological risk assessment: Bridging to population-level effects. Environ. Toxicol. Chem..

[128] Ankley G.T., Bencic D.C., Breen M.S., Collette T.W., Conolly R.B., Denslow N.D., Edwards S.W., Ekman D.R., Garcia-Reyero N., Jensen K.M. (2009). Endocrine disrupting chemicals in fish: Developing exposure indicators and predictive models of effects based on mechanism of action. Aquat. Toxicol..

[129] Eder K.J., Clifford M.A., Hedrick R.P., Köhler H.-R., Werner I. (2008). Expression of immune-regulatory genes in juvenile Chinook salmon following exposure to pesticides and infectious hematopoietic necrosis virus (IHNV). Fish Shellfish Immunol..

[130] Hostetter N.J., Evans A.F., Roby D.D., Collis K., Hawbecker M., Sandford B.P., Thompson D.E., Loge F.J. (2011). Relationship of external fish condition to pathogen prevalence and out-migration survival in juvenile steelhead. Trans. Am. Fish. Soc..

[131] Connon R.E., D'Abronzo L.S., Hostetter N.J., Roby D.D., Evans F.F., Loge F.J., Werner I. (2012). Transcription profiling in environmental diagnostics: Health assessments in Columbia River steelhead (*Oncorhynchus mykiss*). Environ. Sci. Technol..

[132] Miller K.M., Li S., Kaukinen K.H., Ginther N., Hammill E., Curtis J.M., Patterson D.A., Sierocinski T., Donnison L., Pavlidis P. (2011). Genomic signatures predict migration and spawning failure in wild Canadian salmon. Science.

[133] Depledge M.H. (2009). Novel approaches and technologies in pollution assessment and monitoring: A UK perspective. Ocean Coast. Manag..

[134] Blasco C., Picó Y. (2009). Prospects for combining chemical and biological methods for integrated environmental assessment. Trends Anal. Chem..

[135] Ankley G.T., Bennett R.S., Erickson R.J., Hoff D.J., Hornung M.W., Johnson R.D., Mount D.R., Nichols J.W., Russom C.L., Schmieder P.K. (2010). Adverse outcome pathways: A conceptual framework to support ecotoxicology research and risk assessment. Environ. Toxicol. Chem..

[136] Villeneuve D.L., Ankley G.T., Martinović D. (2011). Toxicogenomics Applied to Ecological Risk Assessment. Applications of Toxicogenomics in Safety Evaluation and Risk Assessment.

[137] Weston A.D., Hood L. (2004). Systems biology, proteomics, and the future of health care: Toward predictive, preventative, and personalized medicine. J. Proteome Res..

[138] Kitano H. (2002). Systems biology: A brief overview. Science.

[139] Perkins E.J., Chipman J.K., Edwards S., Habib T., Falciani F., Taylor R., van Aggelen G., Vulpe C., Antczak P., Loguinov A. (2011). Reverse engineering adverse outcome pathways. Environ. Toxicol. Chem..

[140] Geist J. (2011). Integrative freshwater ecology and biodiversity conservation. Ecol. Indicat..

[141] Kellert S.R. (1996). The Value of Life: biological Diversity and Human Society.

[142] Liess M., Beketov M. (2011). Traits and stress: Keys to identify community effects of low levels of toxicants in test systems. Ecotoxicology.

[143] Sanderson H. (2002). Pesticide studies. Environ. Sci. Pollut. Res..

[144] Shaw J.L., Moore M., Kennedy J.H., Hill I.R., Graney R.L., Kennedy J.H., Rodgers J.H. (1994). Design and statistic analysis of field mesocosm studies. Aquatic Mesocosm Studies in Ecological Risk Assessment.

[145] Kennedy J.H., Ammann L.P., Waller W.T., Warren J.E., Hosmer A.J., Cairns S.H., Johnson P.C., Graney R.L. (1999). Using statistical power to optimize sensitivity of analysis of variance designs for microcosms and mesocosms. Environ. Toxicol. Chem..

[146] De Jong F.M.W., Mensink B.J.W.G., Smit C.E., Montforts M.H.M.M. (2005). Evaluation of ecotoxicological field studies for authorization of plant protection products in Europe. Hum. Ecol. Risk Assess.

[147] Van den Brink P.J., Braak C.J.F.T. (1999). Principal response curves: Analysis of time-dependent multivariate responses of biological community to stress. Environ. Toxicol. Chem..

[148] Liess M., Ohe P.C.V.D. (2005). Analyzing effects of pesticides on invertebrate communities in streams. Environ. Toxicol. Chem..

[149] Van den Brink P., Ter Braak C. (2012). Response to “Traits and stress: keys to identify community effects of low levels of toxicants in test systems”. Ecotoxicology.

[150] Liess M., Beketov M. (2012). Rebuttal related to “Traits and Stress: Keys to identify community effects of low levels of toxicants in test systems”. Ecotoxicology.

[151] De Laender F., De Schamphelaere K.A.C., Vanrolleghem P.A., Janssen C.R. (2008). Validation of an ecosystem modeling approach as a tool for ecological effect assessments. Chemosphere.

